# Aminosteroid RM-581 Decreases Cell Proliferation of All Breast Cancer Molecular Subtypes, Alone and in Combination with Breast Cancer Treatments

**DOI:** 10.3390/jcm12134241

**Published:** 2023-06-24

**Authors:** Anna Burguin, Jenny Roy, Geneviève Ouellette, René Maltais, Juliette Bherer, Caroline Diorio, Donald Poirier, Francine Durocher

**Affiliations:** 1 Department of Molecular Medicine, Faculty of Medicine, Université Laval, Québec, QC GIV 0A6, Canada; anna.burguin.1@ulaval.ca (A.B.); genevieve.ouellette@crchudequebec.ulaval.ca (G.O.); juliette.bherer@crchudequebec.ulaval.ca (J.B.); donald.poirier@crchudequebec.ulaval.ca (D.P.); 2 Cancer Research Centre, CHU de Québec-Research Centre, Québec, QC G1R 3S3, Canada; jenny.roy@crchudequebec.ulaval.ca (J.R.); rene.maltais@crchudequebec.ulaval.ca (R.M.); caroline.diorio@crchudequebec.ulaval.ca (C.D.); 3 Laboratory of Medicinal Chemistry, Endocrinology and Nephrology Unit, CHU de Québec-Research Center, Québec, QC G1V 4G2, Canada; 4 Department of Social and Preventive Medicine, Faculty of Medicine, Université Laval, Québec, QC GIV 0A6, Canada

**Keywords:** breast cancer, aminosteroid, triple-negative breast cancer, spheroid, personalized therapy, breast cancer treatment, endoplasmic reticulum stress

## Abstract

Breast cancer (BC) is a heterogenous disease classified into four molecular subtypes (Luminal A, Luminal B, HER2 and triple-negative (TNBC)) depending on the expression of the estrogen receptor (ER), the progesterone receptor (PR) and the human epidermal receptor 2 (HER2). The development of effective treatments for BC, especially TNBC, remains a challenge. Aminosteroid derivative RM-581 has previously shown an antiproliferative effect in multiple cancers in vitro and in vivo. In this study, we evaluated its effect in BC cell lines representative of BC molecular subtypes, including metastatic TNBC. We found that RM-581 has an antiproliferative effect on all BC molecular subtypes, especially on Luminal A and TNBC, in 2D and 3D cultures. The combination of RM-581 and trastuzumab or trastuzumab-emtansine enhanced the anticancer effect of each drug for HER2-positive BC cell lines, and the combination of RM-581 and taxanes (docetaxel or paclitaxel) improved the antiproliferative effect of RM-581 in TNBC and metastatic TNBC cell lines. We also confirmed that RM-581 is an endoplasmic reticulum (EnR)-stress aggravator by inducing an increase in EnR-stress-induced apoptosis markers such as BIP/GRP78 and CHOP and disrupting lipid homeostasis. This study demonstrates that RM-581 could be effective for the treatment of BC, especially TNBC.

## 1. Introduction

Breast cancer (BC) is the second cause of death by cancer in women. The American Cancer Society estimates that 279,100 new cases of invasive BC were diagnosed, and 42,690 deaths were reported among US women in 2020 [1]. BC is a heterogenous disease histologically as well as molecularly. It can be divided into four molecular subtypes: luminal A, luminal B, HER2 and triple-negative breast cancer (TNBC). These four molecular subtypes are determined by the overexpression of three molecular markers: estrogen receptor (ER), progesterone receptor (PR), and the human epidermal receptor 2 (HER2) [2]. Each BC molecular subtype has a different prevalence, prognosis and response to treatment [3]. Due to the complexity of BC, it is challenging to find treatments that can be effective against all BC subtypes. This is especially true for the TNBC subtype for which there are no targeted therapies as this molecular subtype lacks the expression of ER and PR and does not overexpress HER2 [4]. Hence there is an obvious need for the development of novel compounds that could help to effectively target BC. 

Aminosteroid (AM) derivatives are a family of anticancer molecules that have high and selective cytotoxic effects on multiple types of cancers, both in vitro and in vivo [5,6,7,8,9,10,11]. Various AM derivatives have been generated by our group using structure–activity relationship studies involving the parallel synthesis of AM libraries and using classical medicinal chemistry targeting the modification of a specific hit compound [5,7,8,12,13]. Following these analyses, RM-581 has emerged as the most promising candidate. RM-581 is a mestranol derivative with an estra-1,3,5(10)-triene backbone that has been shown to lead to a decrease in cell proliferation and tumor regression in luminal A breast (MCF7), pancreatic (PANC-1), and prostate (PC-3) cancer cell lines as well as xenografts in nude mice [10,11,14].

The endoplasmic reticulum (EnR) is an organelle with a major role in the maintenance of cellular homeostasis [15]. The tumor microenvironment, which is characterized by hypoxia and low-nutrient conditions, can trigger EnR-stress due to the accumulation of unfolded protein in EnR and the related unfolded protein response (UPR). This EnR-stress, in turn, induces cellular dysfunctions that can lead to apoptosis [16]. Natural derivatives such as AM derivatives are anticancer agents that use EnR-stress to induce cancer cell death [17,18,19,20]. It has been recently shown that the RM-581 mechanism of action induces EnR-stress leading to apoptosis in gemcitabine-resistant pancreatic cancer (PANC-1) and docetaxel-resistant prostate cancer (PC-3) cell lines [11,14]. Moreover, many studies have shown that apoptosis via EnR-stress can be targeted in BC, highlighting the importance of this mechanism of action in BC [21,22,23,24]. 

Hence, the aim of the present study was to evaluate whether RM-581 can be an effective anticancer molecule on all BC molecular subtypes (luminal A, luminal B, HER2, and TNBC) and determine if its mechanism of action is via EnR-stress as for pancreatic and prostate cancer. We thus tested RM-581 antiproliferative effects in BC cell lines belonging to different molecular subtypes, alone and in combination with other BC gold standard treatments. We then confirmed these results in 3D BC spheroids and also measured the effect of RM-581 on the expression of EnR-stress markers.

## 2. Materials and Methods

### 2.1. Cell Lines 

We selected BC cell lines representative of luminal A (MCF7), luminal B (BT-474), HER2 (JIMT-1 and MDA-MB-453), TNBC (MDA-MB-231, BT-549, SUM159PT, MDA-MB-468, and SUM149PT), a non-tumorigenic epithelial cell line (MCF10A), and three metastatic TNBC-derivative cell lines (MDA-BoM-1833, MDA-BoM-1834, and MDA-MB-231-BR). All the BC cell lines along with their molecular subtype and the culture conditions are listed in Table S1. All the cell lines were incubated at 37 °C with 5% CO_2_ in water-saturated atmosphere and were shown to be mycoplasma free. 

### 2.2. Proliferation and Drug Combination Assays

Cells were plated on 96-well plates in triplicate (10,000 cells per well) as described in [25]. After 24 h of incubation, RM-581 [10] was diluted in dimethylsulfoxide (DMSO) and added at increasing concentrations (0, 0.1, 1, 10, 20, and 30 µM) in the culture medium. For combination assays, BT-474 and MDA-MB-453 cells received a RM-581 treatment at 10 µM and a trastuzumab (Roche, Genentech, CA, USA) treatment at 4 µg/mL (after dilution of trastuzumab in phosphate buffered saline (PBS)) or a T-DM1 (Roche, Genentech, CA, USA) treatment at 10^−1^ µM (after dilution in PBS) only for MDA-MB-453, alone or in combination. MDA-MB-231, MDA-MB-468, BT-549, SUM149PT, MDA-BoM-1833, MDA-BoM-1834, and MDA-MB-231-BR cells received a RM-581 treatment at 0.1, 1, or 5 µM, a docetaxel (DTX) (Sigma-Aldrich Canada Co., Oakville, ON, Canada, BCBH8742V) and a paclitaxel (PTX) (Sigma-Aldrich Canada Co., Oakville, ON, Canada MKCG8516) treatment at 10−1 µM, 10−2 µM or 10−3 µM (after dilution in DMSO), alone or in combination. After 72 h of in-cubation, alamarBlue (Invitrogen, Waltham, MA, USA, DAL1100) was added (10 µL of alamarBlue for 100 µL of cell culture medium) for 2 h of incubation. Viability rates were determined by fluorescence using a Tecan M-200 microplate reader (Männedorf, Switzerland) with an excitation wavelength at 570 nm and emission wavelength at 585 nm. Percentage viability was calculated for the treated cells compared with the untreated cells receiving only DMSO. All the experiments were performed in triplicate, and mean ± SD was calculated and plotted for each drug concentration. The IC_50_ values (50% cell growth inhibition) for each cell line were calculated using GraphPad software version 5 with a dose-response model (Y = Bottom + (Top-Bottom)/(1 + 10^(X − LogIC50)). For all the curves, the R2 value was greater than 0.9. All the figures were generated using Prism/Graphpad (version 5). For the combination assays, one-way ANOVA were performed followed by the Tukey test to compare the effects of the drug alone or the combination of the drugs. The significance level was set at *p* < 0.05.

### 2.3. Spheroids Assay (3D Culture)

BC cells (MCF7, BT-474, MDA-MB-453, MDA-MB-468, MDA-MB-231, SUM149PT, MCF10A, MDA-BoM-1833, MDA-BoM-1834, and MDA-MB-231-BR) were plated in 96-well ultra-low attachment plates (Corning Costar, NY, USA, 7007) in triplicate (10,000 cells per well). After 5 days of incubation to let the spheroids or cellular aggregates form, RM-581 was added at a concentration twice or five times the IC_50_ obtained in 2D culture, or with 5 µM for MCF10A as a control. Images of the spheroids were taken before, and 3 and 7 days after the treatment to observe phenotype changes. Viability rates were determined as described in the section above. Nonparametric Mann–Whitney tests were performed. The significance level was set at *p* < 0.05. 

### 2.4. RNA Isolation and Quantitative Real-Time PCR (qPCR)

BC cells (MCF7, BT474, MDA-MB-453, MDA-MB-468, MDA-MB-231, BT-549, SUM149PT, MDA-BoM-1833, MDA-BoM-1834, and MDA-MB-231-BR) were plated in 6-well plates (1.5 × 106 cells per well). After 24 h of incubation, RM-581 was added at specific IC50 concentrations for each cell line in a time-dependent manner (0, 3, 6, 12, and 24 h). The cells were homogenized with QIazol Lysis Reagent (QIAGEN, Hilden, Germany, 1023537), and total RNA was extracted following the miRNeasy kit’s instructions (QIAGEN, Hilden, Germany, 1038703). RNA concentrations were measured based on their intrinsic absorptive properties (260/280 and 260/230 ratios) using a Nanodrop ND-1000 Spectrophotometer (NanoDrop Technologies, Wilmington, DE, USA). Reverse transcription was performed using 3 to 5 µg of total RNA. First, total RNA was mixed with 10 mM of dNTP (Invitrogen, Waltham, MA, USA, 18427-013), 50 µM of oligodT (IDT, 272857423) and 50 µM of random hexamers (Invitrogen, Waltham, MA, USA, N8080127) at 65 °C for 5 min. Then, Reverse Transcriptase (Superscript IV, Invitrogen, 18090050, RNaseOut 10777-019) was added for a series of incubations: 10 min at room temperature, 15 min at 50 °C and 10 min at 80 °C. Purification was performed following the QIAquick PCR Purification kit’s instructions (QIAGEN, 28104). cDNA concentration was measured using Nanodrop (260/280 and 260/230 ratios). Real time PCR quantifica-tion was performed in triplicate using 20 ng of cDNA and SYBr Green mix (Thermo Fisher Scientific, Waltham, MA, USA 2010604). 

A melting curve was created to assess non-specific signals. The relative quantity was calculated using the fit point method and by applying the delta cycle threshold (Ct) method as the amplification efficiencies of the curves were 100% [26]. Hypoxanthine guanine phosphoribosyl transferase 1 (*HPRT1*) and glyceraldehyde-3-phosphate dehydrogenase (*GAPDH*) were used as reference genes. To control the genomic DNA contamination in cDNA samples we used a fragment of genomic DNA from the 3β-hydroxysteroid dehydrogenase (*3βHSD*) gene. The mRNA levels are indicated in percentage terms relative to reference genes. The primer sequences are reported in Table S2. Nonparametric Mann–Whitney tests were performed. The significance level was set at *p* < 0.05.

## 3. Results

### 3.1. RM-581 Has an Antiproliferative Effect on Breast Cancer Cell Lines

#### 3.1.1. RM-581 Antiproliferative Effect Found in 2D Culture

To assess the antiproliferative capacity of RM-581, we treated each cell line with a range of concentrations of RM-581 to determine the IC_50_. We first tested a normal breast cell line (MCF10A) and BC cell lines depending on their molecular BC subtype. As shown in Table 1, the IC_50_ of RM-581 (50% of cell growth inhibition) for MCF10A was 17.1 µM, which was the highest IC_50_ found in this study. The graph for each cell line is shown in Figure S1. 

The IC_50_ obtained for all the BC cell lines were significantly different from the IC_50_ obtained from the normal breast cell line MCF10A, except for MDA-MB-453. This result demonstrates that RM-581 is more effective against cancerous cells than against normal cells in the breast. As shown in Table 1, luminal A (ER+/PR+ and HER2-) and TNBC (ER-/PR- and HER2-) cell lines seem to be more sensitive to RM-581, with IC_50_ values of 2.8, 10.4, 8.7, 8.5, 6.9, and 5.6 µM for MCF7, MDA-MB-231, BT-549, SUM159PT, MDA-MB-468, and SUM149PT, respectively. In contrast, Luminal B (ER+/PR+ and HER2+) and HER2 (ER-/PR- and HER2+) cell lines had IC_50_ values of 12.0, 13.4 and 12.3 µM for BT-474, MDA-MB-453 and JIMT-1, respectively. This suggests that the overexpression of HER2 leads to a decrease in the effectiveness of RM-581 against these cell lines. Even though the IC_50_ of MDA-MB-453 and MCF10A are not different, RM-581 could still be effective against HER2 cell lines as the IC_50_ of the other HER2 cell line, JIMT-1, is significantly lower than that of MCF10A. 

Taken together, these results demonstrate that RM-581 was effective on all tested BC cell lines. However, each molecular BC subtype cell line had different sensitivity to RM-581. The luminal A and TNBC cell lines were the most sensitive, while the luminal B and HER2 cell lines were less sensitive.

#### 3.1.2. RM-581 Antiproliferative Effect Found in 3D Culture

**Figure 1:**
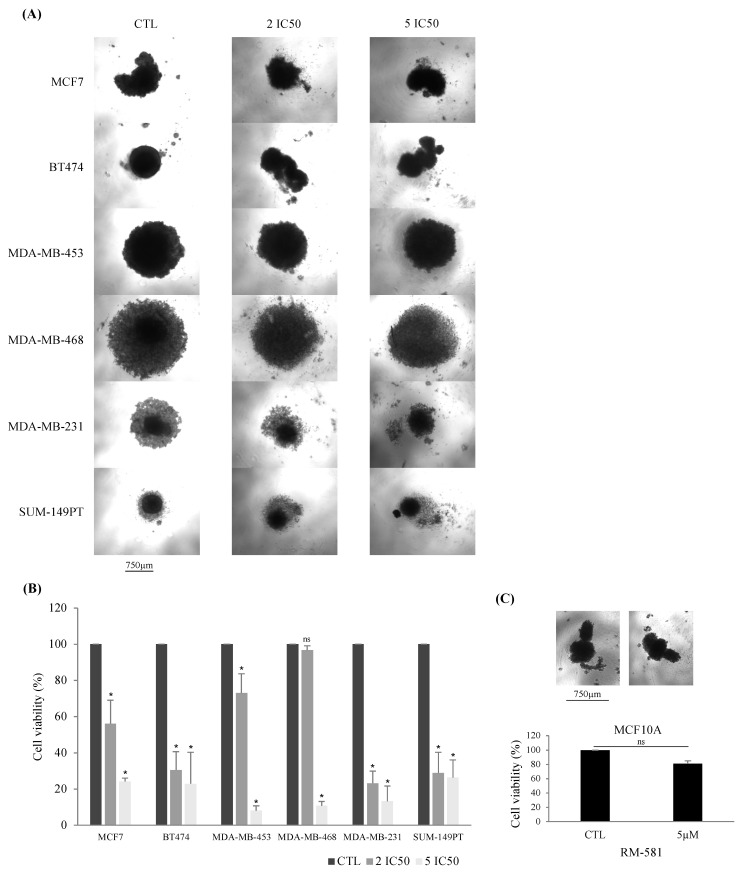
Confirmation of RM-581 efficacy in 3D culture. Spheroids of each cell line were formed using ultra-low attachment plates. RM-581 treatment was administered at 2 or 5 times the concentration of IC_50_ found in 2D culture for each cell line. (**A**) Representative pictures of the spheroids after RM-581 treatment. (**B**) Cell proliferation assay after 7 days of RM-581 treatment. All experiments were performed in triplicate. (**C**) Cell proliferation assay for the normal breast cell line MCF10A with 5 µM of RM-581. Mann–Whitney tests were performed * *p* < 0.05 vs. CTL. ns: non-significant vs. CTL.

We further confirmed the antiproliferative effect of RM-581 in 3D culture, by selecting six BC cell lines (MCF7 (Luminal A), BT-474 (Luminal B), MDA-MB-453 (HER2), MDA-MB-468 (TNBC), MDA-MB-231 (TNBC), and SUM149PT (TNBC)) to perform spheroid assays (Figure 1A). We selected these cell lines to have a representation of each molecular subtype. For the TNBC subtype, we selected the two cell lines with the lowest IC_50_ (MDA-MB-468 and SUM149PT) and the cell line with the highest IC_50_ (MDA-MB-231) to compare the effect of RM-581 within this molecular subtype. Each cell line was treated with two or five times the concentration of IC_50_ determined in the 2D culture. As shown in Figure 1B, two times the concentration of IC_50_ is sufficient to significantly decrease BC cell line spheroid proliferation, except in MDA-MB-468. This could be explained by the fact that the spheroids formed for the MDA-MB-468 cell line were larger in size than in the other BC cell lines, which implies that this cell line proliferates faster (Figure 1A). However, when a concentration of five times the IC_50_ of RM-581 was used, the proliferation significantly decreased for all the BC cell lines spheroids. The percentage viability was around 20% or less for all the BC cell lines, indicating that most cells were dead (Figure 1B). Moreover, the proliferation of the normal breast cell line MCF10A was not affected when treated with 5 µM of RM-581 in 3D culture (Figure 1C). These results corroborate those found in 2D culture, confirming that RM-581 has an antiproliferative effect against all BC molecular subtypes.

### 3.2. RM-581 Is More Effective in Combination with Other Breast Cancer Treatments

#### 3.2.1. RM-581 in Combination with Anti-HER2 Therapies

One of the treatment strategies for BC is to target one of the molecular markers in order to have a personalized treatment for each molecular subtype. Trastuzumab, a well-established BC treatment, is a monoclonal antibody that targets one of the extracellular subdomains of HER2 [27]. A derivative of trastuzumab called T-DM1 (trastuzumab-emtansine) is an antibody drug conjugate formed with trastuzumab and the cytotoxic molecule DM1 [28]. These treatments are administered specifically to patients with HER2+ BC. We evaluated if RM-581 and trastuzumab or T-DM1 could have a greater effect on the decreasing cancer cell proliferation when administered in combination. 

**Figure 2:**
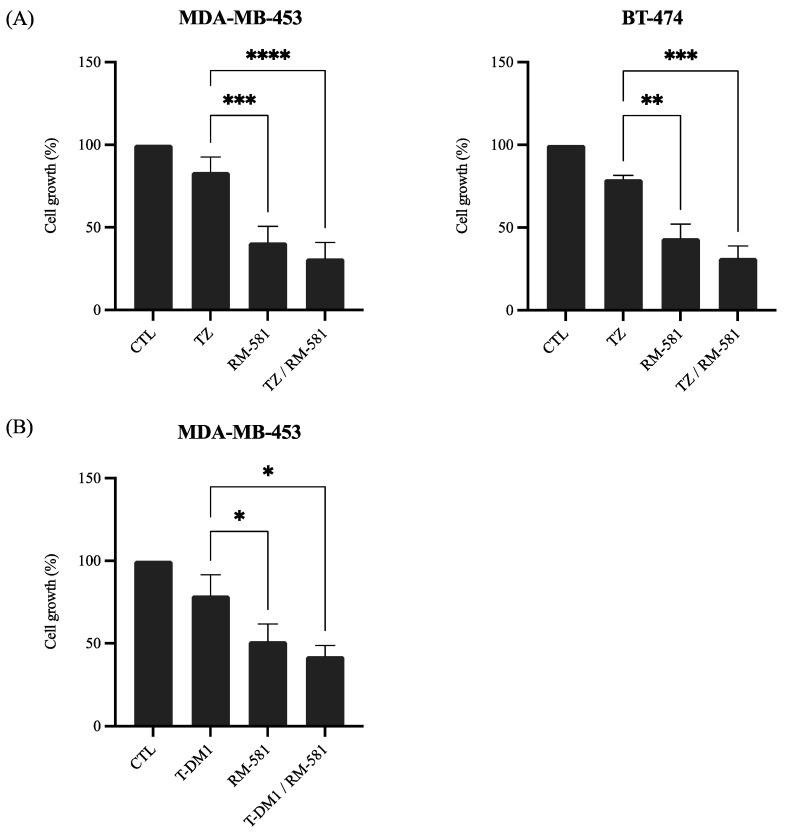
Combination of RM-581 with trastuzumab or T-DM1. Proliferation assays were performed for: (**A**) MDA-MB-453 and BT-474 cells treated with trastuzumab (TZ) at 4 µg/mL and RM-581 at 10 µM; and (**B**) MDA-MB-453 cells treated with trastuzumab-emtansine (T-DM1) at 10^−1^ µM and RM-581 at 10 µM. All experiments were performed in triplicate. One-way ANOVA was performed followed by Tukey test. * *p* < 0.05, ** *p* < 0.01, *** *p* < 0.001, **** *p* < 0.0001.

As shown in Figure 2A, the combination of RM-581 and trastuzumab significantly improved the anticancer effect of trastuzumab alone. The addition of trastuzumab to RM-581 improved the effect compared with RM-581 alone for both HER2 cell lines but not significantly (Figure 2A). As shown in Figure 2B for MDA-MB-453, the combination of T-DM1 and RM-581 significantly improved the effect compared with T-DM1 alone and RM-581 alone but did not significantly decrease cancer cell proliferation. 

Taken together, these results show a benefit of using RM-581 in combination with anti-HER2 therapies to treat HER2+ BC patients effectively.

#### 3.2.2. RM-581 in Combination with Chemotherapy

**Figure 3:**
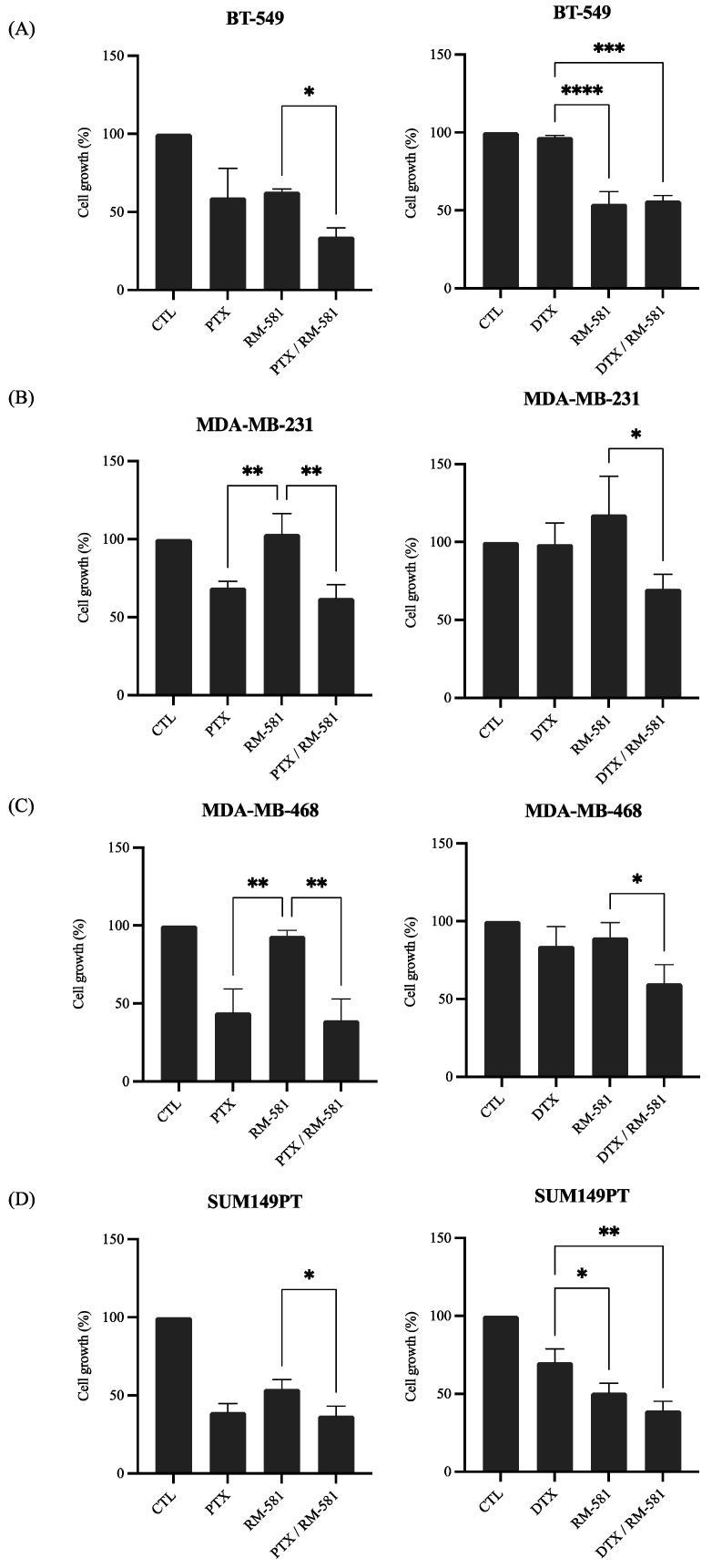
Combination of RM-581 with chemotherapy drugs. Proliferation assays were performed with paclitaxel (PTX) at 10^−3^ µM or docetaxel (DTX) at 10^−2^ µM and RM-581 at 1 µM for BT-549 (**A**) and MDA-MB-231 (**B**), or RM-581 at 0.1 µM for MDA-MB-468 (**C**), or RM-581 at 5 µM for SUM149PT (**D**). Each drug was administered alone and in combination. All experiments were performed in triplicate. One-way ANOVA was performed followed by Tukey test. * *p* < 0.05, ** *p* < 0.01, *** *p* = 0.001, **** *p* < 0.0001.

One of the major strategies of treatment for BC is chemotherapy, which is administered regardless of BC subtypes along with personalized therapy. However, for TNBC, no personalized therapy is currently available, and chemotherapy remains the only pharmaceutical treatment strategy [29]. We evaluated the effect of the combination of RM-581 and PTX or DTX, two taxanes used as chemotherapy treatment, on the proliferation of four TNBC cell lines (BT-549, MDA-MB-231, MDA-MB-468, and SUM149PT). For BT-549, the antiproliferative effect of RM-581 was significantly improved when the drug was administered in combination with PTX. As for DTX, the combination of DTX and RM-581 significantly improved the antiproliferative effect of DTX but not that of RM-581 (Figure 3A). For MDA-MB-231 and MDA-MB-468, when RM-581 was administered with either DTX or PTX, the combination of drugs improved the antiproliferative effect of RM-581 but not that of the taxanes (Figure 3B,C). Finally, for SUM149PT, the combination of RM-581 and PTX significantly improved the antiproliferative effect of RM-581 but not that of PTX. As for DTX, the antiproliferative effect of DTX and RM-581 was significantly improved when the drugs were administered in combination compared with the administration of each drug alone (Figure 3D). 

These results demonstrate that RM-581 could be administered in combination with taxanes as it does not compete with the effects of DTX or PTX on TNBC cell lines. 

### 3.3. RM-581 Increases the Expression of Endoplasmic Reticulum Stress Apoptosis Markers

**Figure 4:**
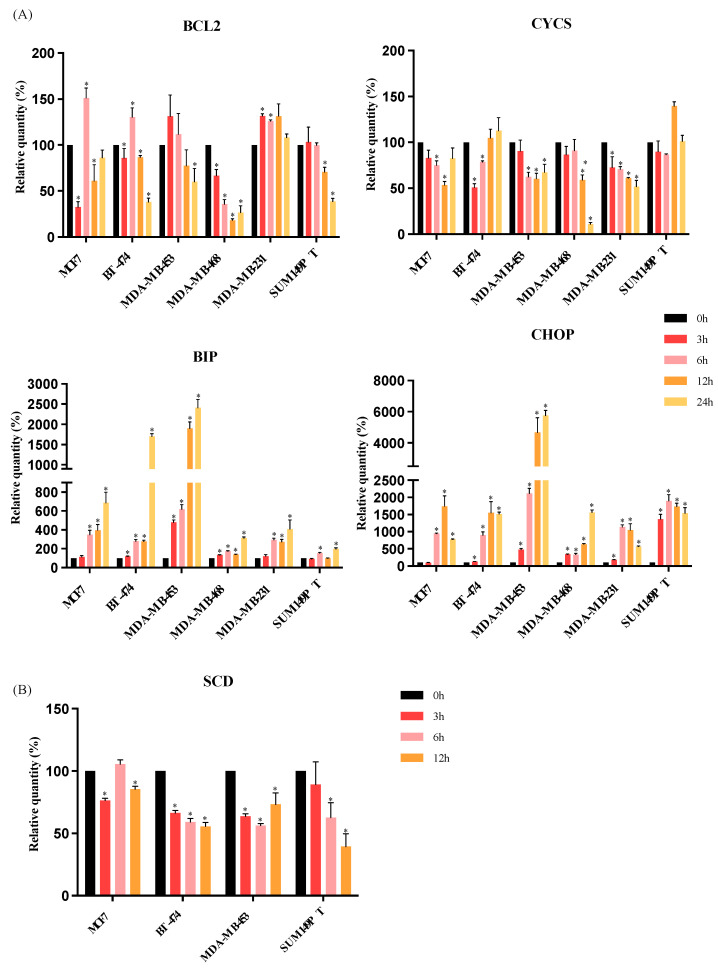
RM-581 induces endoplasmic reticulum stress apoptosis and disturbs lipid synthesis in breast cancer cell lines. (**A**) Each cell line was treated with the specific IC_50_ concentration for different exposure times (0, 3, 6, 12, and 24 h). RT-qPCR transcript quantifications of (**A**) canonical apoptosis pathway markers (*BCL2* and *CYCS*) and endoplasmic reticulum stress apoptosis markers (*BIP* and *CHOP*) and of (**B**) lipid synthesis marker *SCD*. Controls: *GAPDH* and *HPRT1*. Mann–Whitney test was performed. * *p* < 0.05 vs. control (0 h).

The mechanism of action of RM-581 has been previously investigated in pancreatic and prostate cancers as an EnR-stress aggravator [11,14]. Hence, we investigated whether RM-581 anticancer effects lead to EnR-stress apoptosis in BC cells as well. Each BC cell line, i.e., MCF7 (luminal A), BT-474 (luminal B), MDA-MB-453 (HER2), MDA-MB-468 (TNBC), MDA-MB-231 (TNBC), and SUM149PT (TNBC), was treated in a time-dependent manner (0 h, 3 h, 6 h, 12 h, and 24 h) with the IC_50_ concentration established with the proliferation assay to observe the early effects of exposure to RM-581. The mRNA expression levels of intrinsic apoptosis markers (*BCL2* and *CYCS*), EnR-stress marker (*BIP*), and EnR-stress apoptosis marker (*CHOP*) were analyzed. For each of the BC cell lines tested, exposure to RM-581 did not affect the expression of *CYCS* (Figure 4A). The expression of *BCL2* significantly increased after 6 h of RM-581 exposure (for MCF7, BT-474 and MDA-MB-231 cell lines); however, this increase seemed to be transitory as from 12 h, *BCL2* expression significantly decreased for MCF7 and BT-474. On the other hand, the expression of *BIP* and *CHOP* significantly increased in parallel with the exposure time to RM-581 for all BC cell lines (Figure 4A). Moreover, the increase in *BIP* and *CHOP* expression was higher than *BCL2* expression after RM-581 exposure, as the highest relative quantity of *BCL2* was around 150%, whereas the relative quantities of *BIP* and *CHOP* exceeded 200%. These results support the fact that the RM-581 anticancer mechanism of action is dependent on EnR-stress apoptosis in BC, as observed in pancreatic cancer (PANC-1) and prostate cancer (PC-3) cells [11,14]. 

In addition, EnR is also involved in lipid biosynthesis by producing enzymes of this pathway. Among these, SCD (Stearoyl-CoA desaturase 1) is the rate-limiting enzyme of fatty acids synthesis [30]. The level of *SCD* transcripts was, therefore, measured after 3, 6, and 12 h of RM-581 exposure in the MCF7, BT-474, MDA-MB-453, and SUM149PT BC cell lines. As shown in Figure 4B, a decrease in *SCD* expression was observed at the three time points for BT-474 and MDA-MB-453. The *SCD* expression decreased at 3 h and 12 h of RM-581 exposure for MCF7 and for SUM149PT, at 6 h and 12 h of RM-581 exposure. 

Taken together, these results support the fact that RM-581 disrupts lipid homeostasis by inducing EnR-stress-induced apoptosis.

### 3.4. RM-581 Is Effective against TNBC Derivative Metastasis

Metastatic TNBC has the poorest survival rate among the molecular subtypes [31]. Therefore, treatments that could be effective to treat metastatic TNBC are essential and eagerly awaited. We evaluated the efficacy of RM-581 on metastasis derived from TNBC. We selected three cell lines that are subpopulations obtained after inoculation of the MDA-MB-231 cell line into nude mice, which then formed metastasis in their bones (subpopulation 1833), lungs (subpopulation 1834), and in the brain (subpopulation BR) [32,33]. 

**Figure 5:**
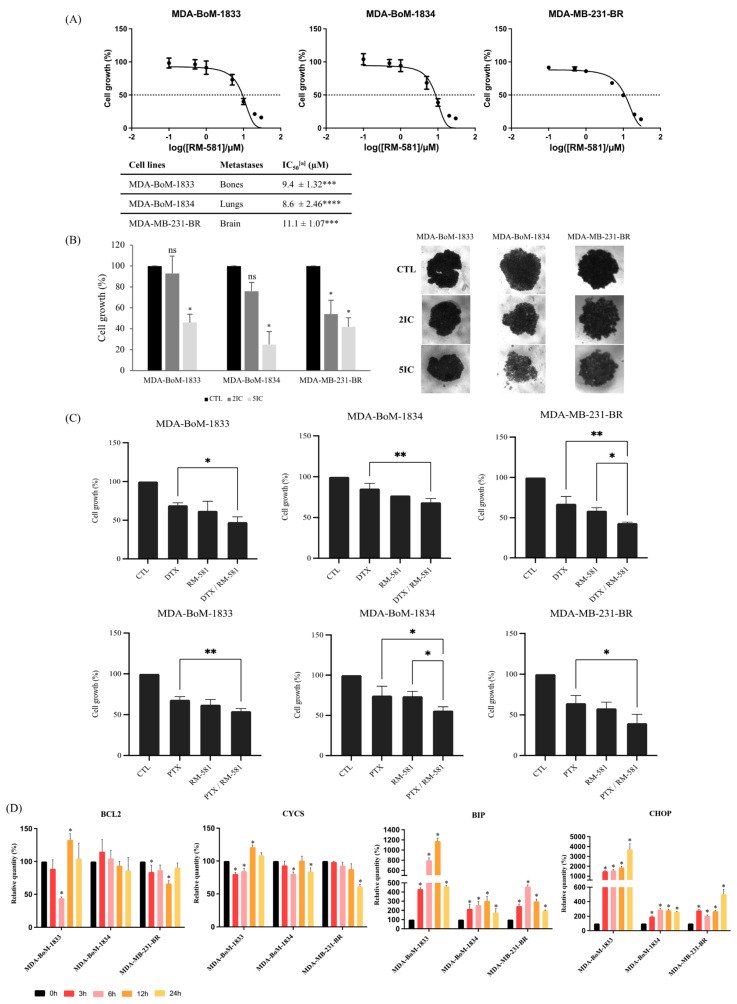
RM-581 is effective against TNBC-derived metastatic cell lines. Three MDA-MB-231-derived metastatic cell lines were selected: MDA-BoM-1833 (bones), MDA-BoM-1834 (lungs), and MDA-MB-231-BR (brain). (**A**) Proliferation assays were performed for each cell line, with increasing doses of RM-581 (0, 0.1, 0.5, 1, 5, 10, 20, and 30 µM). The cell growth was calculated as the percentage of treated cells compared with untreated cells. All experiments were performed in triplicate and means ± SD were calculated and plotted for each drug concentration. Each dot represents the mean value of three experiments ± SD. ^[a]^: means from 3 experiments performed in triplicate ± SD. One-way ANOVA followed by Tukey test were performed. *** *p* < 0.001; **** *p* < 0.0001 compared with the normal cell line MCF10A. (**B**) RM-581 treatments were administered at 2 times or 5 times the concentration of IC_50_ found in 2D culture for each cell line. Cell proliferation assays were performed after 7 days of RM-581 treatment. Representative images of the spheroids are shown. All experiments were performed in triplicate. Mann–Whitney tests were performed. * *p* < 0.05 vs. CTL. ns: non-significant vs. CTL. (**C**) Proliferation assays were performed with DTX or PTX at 10^−1^ µM and RM-581 at 5 µM for each selected cell line. Each drug was administered alone and in combination. All experiments were performed in triplicate. One-way ANOVA followed by Tukey test was performed. * *p* < 0.05 ** *p* < 0.01. (**D**) Each cell line was treated with the specific IC_50_ concentration for different times of exposure (0, 3, 6, 12, and 24 h). RT-qPCR transcript quantifications of canonical apoptosis pathway markers (*BCL2* and *CYCS*) and endoplasmic reticulum stress apoptosis markers (*BIP* and *CHOP*). Controls: *GAPDH* and *HPRT1*. Mann–Whitney was performed. * *p* < 0.05 vs. control (0 h).

The IC_50_ values for the three TNBC metastatic cell lines were 9.4 µM, 8.6 µM, and 11.1 µM for MDA-BoM-1833, MDA-BoM-1834, and MDA-MB-231-BR, respectively (Figure 5A). These values were significantly different from the IC_50_ values found for the normal cell line MCF10A. Thus, RM-581 has an antiproliferative effect on TNBC metastatic cell lines. 

These results were also confirmed in 3D culture by performing a proliferation assay on TNBC metastatic cell line spheroids. As shown in Figure 5B, twice the concentration of the IC_50_ found in 2D culture was sufficient to significantly decrease the proliferation of MDA-MB-231-BR spheroids, but not MDA-BoM-1833 and MDA-BoM-1834 spheroids. However, when 5 times the concentration of the IC_50_ found in 2D culture was applied, the proliferation of all three TNBC metastatic cell lines spheroids significantly decreased below 50% cell viability. 

In addition, for all three TNBC metastatic cell lines, the combination of RM-581 and chemotherapy drug (DTX or PTX) seemed to improve the anticancer effect of each drug alone (Figure 5C). Indeed, the combination of DTX and RM-581 significantly improved the antiproliferative effect of DTX for all three TNBC metastatic cell lines and significantly improved the antiproliferative effect of RM-581 for MDA-MB-231-BR. The combination of PTX and RM-581 significantly improved the antiproliferative effect of PTX for all three TNBC metastatic cell lines, and significantly improved the antiproliferative effect of RM-581 for MDA-BoM-1834. 

The mechanism of action of RM-581 was the same in TNBC metastatic cell lines as in the other BC cell lines used in this study. Indeed, RM-581 treatment did not change the expression of *BCL2* and *CYCS* transcripts for any of the three TNBC metastatic cell lines. In contrast, the level of both *BIP* and *CHOP* transcripts were higher than the control (0 h) for all times of exposure (3, 6, 12, and 24 h) for all three TNBC metastatic cell lines (Figure 5D), indicating that the RM-581 mechanism of action is dependent on EnR-stress-induced apoptosis.

## 4. Discussion

In this study we assessed the anticancer effect of RM-581 in twelve BC cell lines. RM-581 could be a therapeutic strategy to treat BC and even metastatic TNBC as we demonstrated that RM-581 has an antiproliferative effect on all BC molecular subtypes and metastatic TNBC cell lines both in 2D and in 3D cultures. 

Our results are concordant with a previous study where RM-581 effects were tested in the luminal A (MCF7) BC cell line [10]. We found that the IC_50_ of RM-581 for the BC cell line MCF7 was 2.8 µM and was 17.1 µM for MCF10A, which is very similar to the results found by Perreault et al. [10]. However, they only focused on one BC molecular subtype, luminal A, whereas our study has demonstrated the effectiveness of RM-581 on all BC molecular subtypes (luminal A, luminal B, HER2, and TNBC) as well as TNBC derived metastatic cell lines. 

We have shown that RM-581 displays the potential to treat patients with TNBC. For the TNBC cell lines, the mean IC_50_ was around 8 µM. RM-581 is particularly effective against MDA-MB-468 (6.9 µM) and SUM149PT (5.6 µM) compared with MDA-MB-231 (10.4 µM), BT-549 (8.7 µM), and SUM159PT (8.5 µM). This difference in sensitivity could be explained by different TNBC subtypes; MDA-MB-468 and SUM149PT are basal, and MDA-MB-231, BT-549 and SUM159PT are of the mesenchymal TNBC subtype, according to the classification of Lehmann et al. [34]. It is known that the basal-like TNBC subtype is highly proliferative due to an enrichment of genes involved in the proliferation pathway [34]. RM-581 seems to target proliferative cells as it is less harmful to normal cell line MCF10A. 

Drug combination is an important part of BC treatments and regimens as it is a very heterogeneous disease. Combination treatments can prevent BC patients from side effects, drug resistance and recurrence [35]. Trastuzumab is a neoadjuvant/adjuvant therapy that sensitizes cancer cells to other treatment. It is well known that trastuzumab does not have a drastic effect on inhibiting tumor growth [25,36,37]. A similar effect was observed for RM-581 on HER2-positive BC cell lines. We demonstrated that the combination of trastuzumab and RM-581 significantly improves the efficacy of both drugs in BT-474, and significantly improves the efficacy of trastuzumab in MDA-MB-453, with an enhancement of RM-581 efficacy. On the other hand, T-DM1 is a treatment administered to patients with HER2+ advanced BC [38]. We found similar effects to the combination of trastuzumab and RM-581 with the combination of T-DM1 and RM-581 on MDA-MB-453. We showed that HER2-positive BC cell lines have the highest IC_50_ for RM-581 compared with the other molecular subtypes. Thus, the combination of trastuzumab or T-DM1 and RM-581 could lead to a reduction in the RM-581 concentration administered to patients with HER2-positive BC. 

Due to the lack of ER and PR expression as well as HER2 overexpression, patients with TNBC cannot receive targeted therapies such as hormonal therapy or anti-HER2 therapy, and chemotherapy remains the only treatment for these patients [39]. Long-term effects of chemotherapy include cardiomyopathy, second cancers, early menopause, sterility, and psychosocial impacts [29]. Here, we demonstrated that the combination of DTX or PTX with RM-581 increases the antiproliferative effects of the taxanes compared with the administration of the drug alone on all four TNBC cell lines. The combination of RM-581 with chemotherapy drugs such as taxanes could be used to treat patients with TNBC more efficiently and reduce the concentration of DTX and PTX to avoid long-term side effects. 

Although the survival rate of BC patients is encouraging with a 5 year relative cancer-specific survival rate of 90.3%, the survival rate of metastatic BC patients remains low, especially for patients with metastatic TNBC, with a 5 year relative cancer-specific survival rate of 12% [40]. Thus, it is crucial to find new solutions for the treatment of metastatic BC. We used the three cell lines MDA-BoM-1833, MDA-BoM-1834, and MDA-MB-231-BR as models for metastatic TNBC. These cell lines are good models as they maintain their metastatic activity after multiple passages and they show a distinct transcriptional signature depending on their metastatic site [32,33,41]. We demonstrated that RM-581 is effective against all three metastatic TNBC cell lines, representing a potential hope for patients with metastatic TNBC. 

The EnR is the organelle responsible for the production and folding of cellular proteins and contributes to cell homeostasis. Due to extracellular environmental disturbances, unfolded proteins can accumulate in the EnR lumen, causing the activation of a specific stress pathway named the unfolded protein response (UPR). The UPR leads to two signaling pathways: pro-survival to restore the cell homeostasis or pro-death if EnR-stress is prolonged [42]. The UPR appears to be an important mechanism maintaining tumor cell malignancy [43]. Therefore, targeting UPR signaling is a new promising therapeutic approach.

Our results support that EnR-stress-induced apoptosis could be involved in the RM-581 mechanism of action. In fact, a gradual increase in *BIP* was observed for all the molecular subtype BC cell lines. BIP (also known as GRP78) is one of the EnR chaperones which are essential to maintain the normal function of the EnR and the activation of the UPR [44,45]. This suggests that RM-581 might induce EnR-stress by activating UPR signaling. Moreover, an increase in *CHOP* with the highest peak at 12 h, was observed for MCF7, BT-474, MDA-MB-231, and SUM149PT and at 24 h for MDA-MB-453 and MDA-MD-468. CHOP is one the UPR downstream effectors and is the principal activator of the apoptosis induced by EnR-stress [46]. Taken together, these results suggest that RM-581 is an EnR-stress aggravator that could induce the EnR-stress-induced apoptosis. This is concordant with previous results observed in pancreatic cancer and prostate cancer cell lines [11,14]. This possible mechanism is in accordance with a previous study showing that RM-581 accumulates in the EnR, indicating that RM-581 could directly interact with an EnR protein and cause EnR-stress-induced apoptosis [14,47].

The EnR is the site of lipid biogenesis, such as fatty acids. Moreover, in cancer cells, the lipid metabolism is affected, which promotes tumor cell survival [48]. Thus, we selected the marker of fatty acid biosynthesis, SCD, to investigate the effect of RM-581 on lipid homeostasis. After the treatment of RM-581 for 12 h, we observed a decrease in *SCD* transcript for all molecular subtype BC cell lines. This suggests that RM-581 could also decrease BC cell proliferation by modulating lipid biosynthesis. Further investigations are needed to decipher the potential mechanism of action of RM-581. 

EnR-stress and UPR signaling are important in tumorigenesis and lead to treatment resistance. This is one of the major challenges for BC therapy as many patients acquire resistance to their treatment [49].Therefore, having new treatments that could target UPR signaling to bypass treatment resistance is a crucial need. RM-581 has been proven to have an anticancer effect in an induced DTX-resistant prostate cancer cell line [14].We have also shown that RM-581 inhibits the proliferation of JIMT-1 (IC_50_ = 12.3 µM), a well characterized trastuzumab-resistant cell line [50].

In this study, we demonstrated that RM-581 is particularly effective against TNBC cell lines, and especially basal-like subtypes (MDA-MB-468 and SUM149PT). Here we tested four of the six TNBC subtypes: BL1 (MDA-MB-468), BL2 (SUM149PT), M (BT-549), and MSL (SUM159PT and MDA-MB-231) subtypes [34]. It would be interesting to test the effect of RM-581 on the two remaining subtypes (IM and LAR) to highlight which TNBC subtype patients could benefit the most from RM-581. 

Spheroids are aggregates of cells grown in suspension that are used as 3D culture models. Multicellular tumor spheroids (MCTS) represent avascular tumor nodules with a transcript profile closer to in vivo tumor gene expression profiles than tumor cells in 2D culture. MCTS are widely used to assess tumor response to new synthetized drugs [51]. We demonstrated that RM-581 is able to decrease the proliferation of tumor cells in 3D for all the BC molecular subtypes, suggesting that RM-581 could be effective in vivo. Obviously, further in vivo tests such as xenografts are required to assess the efficacy and safety of RM-581 in animal models.

## Figures and Tables

**Table 1:** Antiproliferative effect of RM-581 on all BC molecular subtypes cell lines.

Cell Lines	Molecular Subtype	IC_50_ (µM) ^[a]^
MCF10A	Normal	17.1 ± 0.61
MCF7	Luminal A	2.8 ± 1.15 ****
BT-474	Luminal B	12.0 ± 4.28 **
MDA-MB-453	HER2	13.4 ± 0.97
JIMT-1	HER2	12.3 ± 2.21 *
MDA-MB-231	TNBC	10.4 ± 2.40 ***
BT-549	TNBC	8.7 ± 0.37 ****
SUM159PT	TNBC	8.5 ± 1.10 ****
MDA-MB-468	TNBC	6.9 ± 1.03 ****
SUM149PT	TNBC	5.6 ± 0.96 ****

HER2: human epidermal growth factor receptor 2. TNBC: triple-negative breast cancer. ^[a]^: means from 3 experiments performed in triplicate ± SD. One-way ANOVA followed by Tukey tests was performed. * *p* < 0.05; ** *p* < 0.01; *** *p* < 0.001; **** *p* < 0.0001 compared with the normal cell line MCF10A.

## Data Availability

The data presented in this study are available on request from the corresponding author. The data are not publicly available due to privacy.

## Supplementary Materials

The following supporting information can be downloaded at: https://www.mdpi.com/article/10.3390/jcm12134241/s1. Figure S1: RM-581 has an antiproliferative effect on BC cell lines. Proliferation assays were performed for each cell line, with increasing doses of RM-581 (0, 0.1, 1, 10, and 30 µM). The cell growth was calculated as the percentage of treated cells compared with untreated cells. All experiments were performed in triplicate and means ± SD were calculated and plotted for each drug concentration. Table S1: Breast cancer cell lines by molecular subtypes and culture media. ER—estrogen receptor. PR—progesterone receptor. HER2—human epidermal growth factor receptor 2. TNBC—triple-negative breast cancer. Table S2: qPCR primers details.

## References

[1] Siegel R.L., Miller K.D., Jemal A. (2020). Cancer Statistics, 2020. CA Cancer J. Clin..

[2] Harbeck N., Gnant M. (2017). Breast Cancer. Lancet.

[3] Howlader N., Cronin K.A., Kurian A.W., Andridge R. (2018). Differences in Breast Cancer Survival by Molecular Subtypes in the United States. Cancer Epidemiol. Biomark. Prev..

[4] Bianchini G., Balko J.M., Mayer I.A., Sanders M.E., Gianni L. (2016). Triple-Negative Breast Cancer: Challenges and Opportunities of a Heterogeneous Disease. Nat. Rev. Clin. Oncol..

[5] Maltais R., Hospital A., Delhomme A., Roy J., Poirier D. (2014). Chemical Synthesis, NMR Analysis and Evaluation on a Cancer Xenograft Model (HL-60) of the Aminosteroid Derivative RM-133. Steroids.

[6] Talbot A., Maltais R., Poirier D. (2012). New Diethylsilylacetylenic Linker for Parallel Solid-Phase Synthesis of Libraries of Hydroxy Acetylenic Steroid Derivatives with Improved Metabolic Stability. ACS Comb. Sci..

[7] Jegham H., Maltais R., Roy J., Doillon C., Poirier D. (2012). Biological Evaluation of a New Family of Aminosteroids That Display a Selective Toxicity for Various Malignant Cell Lines. Anticancer Drugs.

[8] Kenmogne L.C., Ayan D., Roy J., Maltais R., Poirier D. (2015). The Aminosteroid Derivative RM-133 Shows In Vitro and In Vivo Antitumor Activity in Human Ovarian and Pancreatic Cancers. PLoS ONE.

[9] Maltais R., Perreault M., Roy J., Poirier D. (2020). Minor Chemical Modifications of the Aminosteroid Derivative RM-581 Lead to Major Impact on Its Anticancer Activity, Metabolic Stability and Aqueous Solubility. Eur. J. Med. Chem..

[10] Perreault M., Maltais R., Roy J., Dutour R., Poirier D. (2017). Design of a Mestranol 2-*N*-Piperazino-Substituted Derivative Showing Potent and Selective in Vitro and in Vivo Activities in MCF-7 Breast Cancer Models. ChemMedChem.

[11] Perreault M., Maltais R., Roy J., Picard S., Popa I., Bertrand N., Poirier D. (2019). Induction of Endoplasmic Reticulum Stress by Aminosteroid Derivative RM-581 Leads to Tumor Regression in PANC-1 Xenograft Model. Investig. New Drugs.

[12] Roy J., Maltais R., Jegham H., Poirier D. (2011). Libraries of 2β-(N-Substituted Piperazino)-5α-Androstane-3α, 17β-Diols: Chemical Synthesis and Cytotoxic Effects on Human Leukemia HL-60 Cells and on Normal Lymphocytes. Mol. Divers..

[13] Ayan D., Maltais R., Hospital A., Poirier D. (2014). Chemical Synthesis, Cytotoxicity, Selectivity and Bioavailability of 5α-Androstane-3α,17β-Diol Derivatives. Bioorg. Med. Chem..

[14] Maltais R., Roy J., Perreault M., Sato S., Lévesque J.-C., Poirier D. (2021). Induction of Endoplasmic Reticulum Stress-Mediated Apoptosis by Aminosteroid RM-581 Efficiently Blocks the Growth of PC-3 Cancer Cells and Tumors Resistant or Not to Docetaxel. Int. J. Mol. Sci..

[15] Schröder M., Kaufman R.J. (2005). The Mammalian Unfolded Protein Response. Annu. Rev. Biochem..

[16] Riha R., Gupta-Saraf P., Bhanja P., Badkul S., Saha S. (2017). Stressed Out—Therapeutic Implications of ER Stress Related Cancer Research. Oncomedicine.

[17] Cragg G.M., Grothaus P.G., Newman D.J. (2009). Impact of Natural Products on Developing New Anti-Cancer Agents. Chem. Rev..

[18] Kelloff G.J., Crowell J.A., Steele V.E., Lubet R.A., Malone W.A., Boone C.W., Kopelovich L., Hawk E.T., Lieberman R., Lawrence J.A. (2000). Progress in Cancer Chemoprevention: Development of Diet-Derived Chemopreventive Agents. J. Nutr..

[19] Kim C., Song H.-S., Park H., Kim B. (2018). Activation of ER Stress-Dependent MiR-216b Has a Critical Role in Salvia Miltiorrhiza Ethanol-Extract-Induced Apoptosis in U266 and U937 Cells. Int. J. Mol. Sci..

[20] Cha J., Song H.-S., Kang B., Park M., Park K., Kim S.-H., Shim B.-S., Kim B. (2018). MiR-211 Plays a Critical Role in Cnidium Officinale Makino Extract-Induced, ROS/ER Stress-Mediated Apoptosis in U937 and U266 Cells. Int. J. Mol. Sci..

[21] Clarke R., Cook K.L., Hu R., Facey C.O.B., Tavassoly I., Schwartz J.L., Baumann W.T., Tyson J.J., Xuan J., Wang Y. (2012). Endoplasmic Reticulum Stress, the Unfolded Protein Response, Autophagy, and the Integrated Regulation of Breast Cancer Cell Fate. Cancer Res..

[22] Ko E.-Y., Moon A. (2015). Natural Products for Chemoprevention of Breast Cancer. J. Cancer Prev..

[23] Kamiya T., Nishihara H., Hara H., Adachi T. (2012). Ethanol Extract of Brazilian Red Propolis Induces Apoptosis in Human Breast Cancer MCF-7 Cells through Endoplasmic Reticulum Stress. J. Agric. Food Chem..

[24] Shi J.-M., Bai L.-L., Zhang D.-M., Yiu A., Yin Z.-Q., Han W.-L., Liu J.-S., Li Y., Fu D.-Y., Ye W.-C. (2013). Saxifragifolin D Induces the Interplay between Apoptosis and Autophagy in Breast Cancer Cells through ROS-Dependent Endoplasmic Reticulum Stress. Biochem. Pharmacol..

[25] Burguin A., Furrer D., Ouellette G., Jacob S., Diorio C., Durocher F. (2020). Trastuzumab Effects Depend on HER2 Phosphorylation in HER2-Negative Breast Cancer Cell Lines. PLoS ONE.

[26] Pfaffl M.W. (2001). A New Mathematical Model for Relative Quantification in Real-Time RT-PCR. Nucleic Acids Res..

[27] Paik S., Kim C., Wolmark N. (2008). *HER2* Status and Benefit from Adjuvant Trastuzumab in Breast Cancer. N. Engl. J. Med..

[28] Lewis Phillips G.D., Li G., Dugger D.L., Crocker L.M., Parsons K.L., Mai E., Blättler W.A., Lambert J.M., Chari R.V.J., Lutz R.J. (2008). Targeting HER2-Positive Breast Cancer with Trastuzumab-DM1, an Antibody-Cytotoxic Drug Conjugate. Cancer Res..

[29] Burguin A., Diorio C., Durocher F. (2021). Breast Cancer Treatments: Updates and New Challenges. J. Pers. Med..

[30] Koeberle A., Löser K., Thürmer M. (2016). Stearoyl-CoA Desaturase-1 and Adaptive Stress Signaling. Biochim. Biophys. Acta.

[31] Bergin A.R.T., Loi S. (2019). Triple-Negative Breast Cancer: Recent Treatment Advances. F1000Research.

[32] Kang Y., Siegel P.M., Shu W., Drobnjak M., Kakonen S.M., Cordón-Cardo C., Guise T.A., Massagué J. (2003). A Multigenic Program Mediating Breast Cancer Metastasis to Bone. Cancer Cell.

[33] Dun M.D., Chalkley R.J., Faulkner S., Keene S., Avery-Kiejda K.A., Scott R.J., Falkenby L.G., Cairns M.J., Larsen M.R., Bradshaw R.A. (2015). Proteotranscriptomic Profiling of 231-BR Breast Cancer Cells: Identification of Potential Biomarkers and Therapeutic Targets for Brain Metastasis. Mol. Cell. Proteom. MCP.

[34] Lehmann B.D., Bauer J.A., Chen X., Sanders M.E., Chakravarthy A.B., Shyr Y., Pietenpol J.A. (2011). Identification of Human Triple-Negative Breast Cancer Subtypes and Preclinical Models for Selection of Targeted Therapies. J. Clin. Investig..

[35] Fisusi F.A., Akala E.O. (2019). Drug Combinations in Breast Cancer Therapy. Pharm. Nanotechnol..

[36] Wuerstlein R., Harbeck N. (2017). Neoadjuvant Therapy for HER2-Positive Breast Cancer. Rev. Recent Clin. Trials.

[37] Minckwitz G., Procter M., Azambuja E., Zardavas D., Benyunes M., Viale G., Suter T., Arahmani A., Rouchet N., Clark E. (2017). Adjuvant Pertuzumab and Trastuzumab in Early HER2-Positive Breast Cancer. N. Engl. J. Med..

[38] Amiri-Kordestani L., Blumenthal G.M., Xu Q.C., Zhang L., Tang S.W., Ha L., Weinberg W.C., Chi B., Candau-Chacon R., Hughes P. (2014). FDA Approval: Ado-Trastuzumab Emtansine for the Treatment of Patients with HER2-Positive Metastatic Breast Cancer. Clin. Cancer Res. Off. J. Am. Assoc. Cancer Res..

[39] Wahba H.A., El-Hadaad H.A. (2015). Current Approaches in Treatment of Triple-Negative Breast Cancer. Cancer Biol. Med..

[40] Female Breast Cancer Subtypes—Cancer Stat Facts. https://seer.cancer.gov/statfacts/html/breast-subtypes.html.

[41] Minn A.J., Gupta G.P., Siegel P.M., Bos P.D., Shu W., Giri D.D., Viale A., Olshen A.B., Gerald W.L., Massagué J. (2005). Genes That Mediate Breast Cancer Metastasis to Lung. Nature.

[42] Sisinni L., Pietrafesa M., Lepore S., Maddalena F., Condelli V., Esposito F., Landriscina M. (2019). Endoplasmic Reticulum Stress and Unfolded Protein Response in Breast Cancer: The Balance between Apoptosis and Autophagy and Its Role in Drug Resistance. Int. J. Mol. Sci..

[43] Lee A.S. (2007). GRP78 Induction in Cancer: Therapeutic and Prognostic Implications. Cancer Res..

[44] Wang M., Wey S., Zhang Y., Ye R., Lee A.S. (2009). Role of the Unfolded Protein Response Regulator GRP78/BiP in Development, Cancer, and Neurological Disorders. Antioxid. Redox Signal..

[45] Lee A.S. (2005). The ER Chaperone and Signaling Regulator GRP78/BiP as a Monitor of Endoplasmic Reticulum Stress. Methods.

[46] Marciniak S.J., Yun C.Y., Oyadomari S., Novoa I., Zhang Y., Jungreis R., Nagata K., Harding H.P., Ron D. (2004). CHOP Induces Death by Promoting Protein Synthesis and Oxidation in the Stressed Endoplasmic Reticulum. Genes Dev..

[47] Maltais R., Roy J., Poirier D. (2021). Turning a Quinoline-Based Steroidal Anticancer Agent into Fluorescent Dye for Its Tracking by Cell Imaging. ACS Med. Chem. Lett..

[48] Santos C.R., Schulze A. (2012). Lipid Metabolism in Cancer. FEBS J..

[49] Cosentino G., Plantamura I., Tagliabue E., Iorio M.V., Cataldo A. (2021). Breast Cancer Drug Resistance: Overcoming the Challenge by Capitalizing on MicroRNA and Tumor Microenvironment Interplay. Cancers.

[50] Tanner M., Kapanen A.I., Junttila T., Raheem O., Grenman S., Elo J., Elenius K., Isola J. (2004). Characterization of a Novel Cell Line Established from a Patient with Herceptin-Resistant Breast Cancer. Mol. Cancer Ther..

[51] Friedrich J., Seidel C., Ebner R., Kunz-Schughart L.A. (2009). Spheroid-Based Drug Screen: Considerations and Practical Approach. Nat. Protoc..

