# Supplementary material for: Aminosteroid RM-581 Decreases Cell Proliferation of All Breast Cancer Molecular Subtypes, Alone and in Combination with Breast Cancer Treatments
Source: J Clin Med. 2023 Jun 24;12(13):4241. doi: 10.3390/jcm12134241 (PMC10342875; doi:10.3390/jcm12134241)
Supplement: Supplementary file 1 [file jcm-12-04241-s001.zip › jcm-2426289-supplementary.pdf]

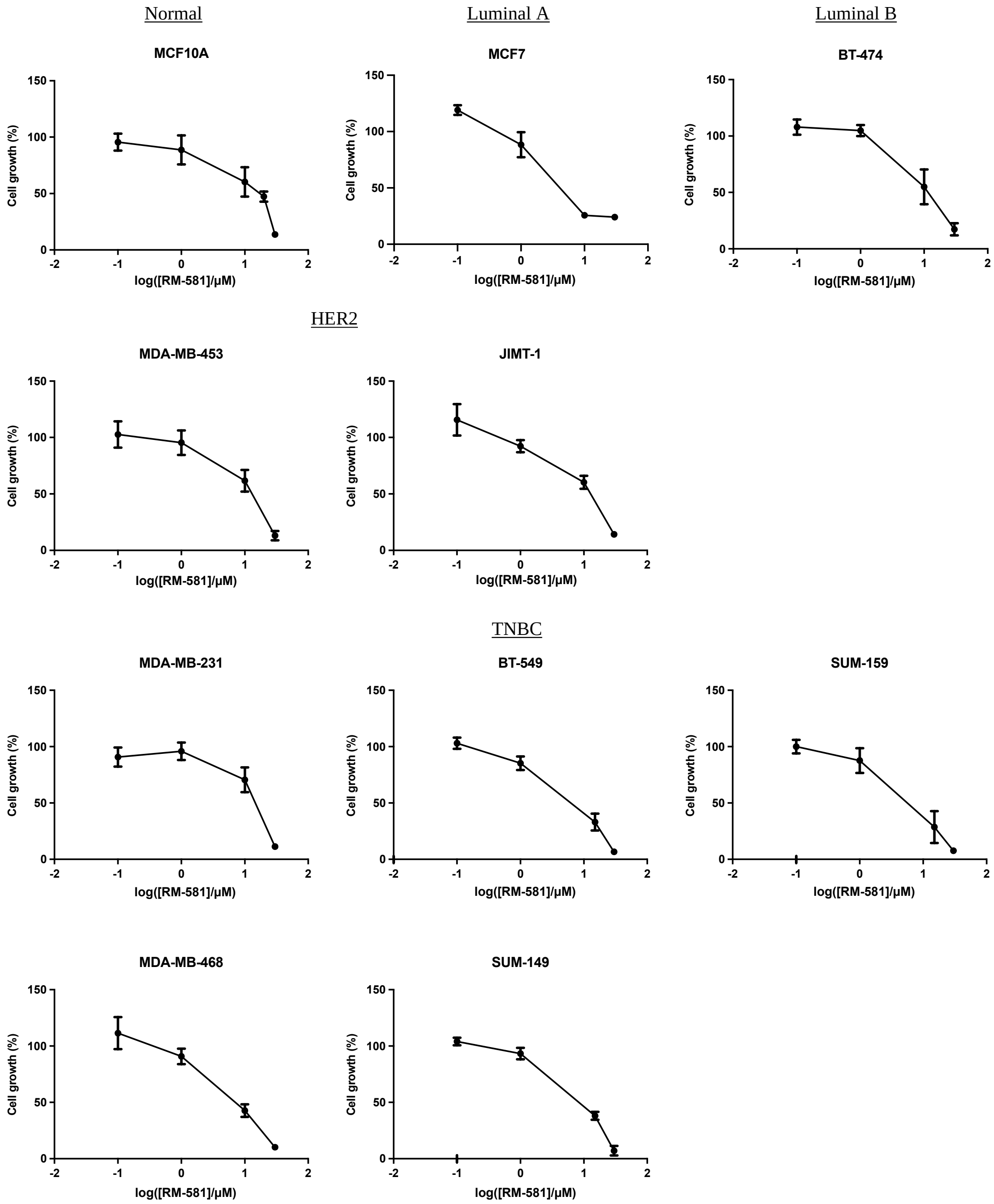

Figure S1. RM-581 has an antiproliferative effect on BC cell lines. Proliferation assays have been performed for each cell line, treated with increasing doses of RM-581 (0, 0.1, 1, 10, and 30μM). The cell growth was calculated as the percentage of treated cells compared to untreated cells. All experiments were done in triplicates and means ± SD were calculated and plotted for each drug concentration.

Table S1. Breast cancer cell lines by molecular subtypes and culture media. ER – Estrogen receptor. PR – Progesterone receptor. HER2 – Human epidermal growth factor receptor 2. TNBC – Triple Negative Breast cancer.

Cell lines	ER status	PR status	HER2 status	Molecular subtypes	Medium	Provenance
MCF7	+	+	-	Luminal A	DMEM F12 (Wisent 319-080) with 5% FBS, 1% Penstrep, 13.4ml sodium bicarbonate 7.5% solution (Wisent 609-105), 7.5ml HEPES 1M (Wisent 330-050), and 10mM estradiol (E2) (Sigma E8875).	American Type Culture Collection (ATCC) - HTB-22
BT-474	+	+	+	Luminal B	RPMI-1640 (Wisent, 350-000-CL) with 10% fetal bovine serum (FBS) (Wisent, 080-150), 1% penicillin-streptomycin (Penstrep) (Wisent, 450-200-EL), and 10μg/ml insulin (Wisent 521-016).	ATCC - HTB-20
BT-549	-	-	-	TNBC		ATCC - HTB-122
JIMT-1	-	-	+	HER2	DMEM (Wisent, 319-005-CL) with 10% FBS and 2mM L-glutamine.	Dr. Marcel B Bally, University of British Columbia, Vancouver, Canada
MDA-MB-453	-	-	+	HER2	RPMI-1640 with 10% FBS and 1% Penstrep.	ATCC - HTB-131
MDA-MB-231	-	-	-	TNBC		ATCC - HTB-26
MDA-MB-468	-	-	-	TNBC		ATCC - HTB-132
SUM149PT	-	-	-	TNBC		BIO IVT
SUM159PT	-	-	-	TNBC		BIO IVT
MDA-BoM-1833	-	-	-	TNBC	DMEM (Wisent 319-005) with 10% FBS and 1% Penstrep.	Dr. Joan Massague Howard Hughes Medical Institute, Memorial Sloan Kettering, New York, NY, USA
MDA-BoM-1834	-	-	-	TNBC		Dr. Joan Massague Howard Hughes Medical Institute, Memorial Sloan Kettering, New York, NY, USA
MDA-MB-231-BR	-	-	-	TNBC		Dr. Patricia S. Steeg National Cancer Institute, Bethesda, MD, USA

Table S2. qPCR primers details.

**Genes**	**Forward sequence** 5'→3'	**Reverse sequence** 5'→3'
**BCL2**	GGTGGGGTCATGTGTGTGGAGAG	TGCAGGTGCCGGTTCAGGTACT
**CYCS**	AACAAAGGCATCATCTGGGGA	AGGCAGTGGCCAATTATTACTCA
**BIP**	CTTGGTATTGAAACTGTGGGAGGTG	TTCCAGTCAGATCAAATGTACCCAG
**CHOP**	GGAGGAGCCAGAACCAGCAGA	TTCCGTTTCCTGGTTCTCCCTT
**GAPDH**	GGCTCTCCAGAACATCATCCCT	ACGCCTGCTTCACCACCTTCTT
**SCD**	CCCACCTCTTCGGATATCGTC	TTGTGGAAGCCCTCACCCAC
**HPRT1**	AGTTCTGTGGCCATCTGCTTAGTAG	AAACAACAATCCGCCCAAAGG
**3βHSD (gDNA)**	GAAGGGCAGAGGTGGAACTAGAA	AACAAAGACCAAAGACCAGTGAGA
